# Subnational regional inequality in the public health development index in Indonesia

**DOI:** 10.1080/16549716.2018.1500133

**Published:** 2018-09-17

**Authors:** Suparmi, Nunik Kusumawardani, Devaki Nambiar, Trihono, Ahmad Reza Hosseinpoor

**Affiliations:** a National Institute of Health Research and Development, Ministry of Health, Jakarta, Republic of Indonesia; b George Institute for Global Health, New Delhi, India; c Health Policy Unit, Ministry of Health, Jakarta, Republic of Indonesia; d Information, Evidence and Research, World Health Organization, Geneva, Switzerland

**Keywords:** Inequality, composite index, public health service, health outcomes, Indonesia

## Abstract

**Background**: Achieving the Sustainable Development Goal of ‘ensuring healthy lives and promoting well-being for all at all ages’ necessitates regular monitoring of inequality in the availability of health-related infrastructure and access to services, and in health risks and outcomes.

**Objectives**: To quantify subnational regional inequality in Indonesia using a composite index of public health infrastructure, services, behavioural risk factors and health outcomes: the Public Health Development Index (PHDI).

**Methods**: PHDI is a composite index of 30 public health indicators from across the life course and along the continuum of care. An overall index and seven topic-specific sub-indices were calculated using data from the 2013 Indonesian Basic Health Survey (RISKESDAS) and the 2011 – Village Potential Survey (PODES). These indices were analysed at the national, province and district levels. Within-province inequality was calculated using the Weighted Index of Disparity (IDISW).

**Results**: National average PHDI overall index was 54.0 (out of a possible 100); scores differed between provinces, ranging from 43.9 in Papua to 65.0 in Bali. Provinces in western regions of Indonesia tended to have higher overall PHDI scores compared to eastern regions. Large variations in province averages were observed for the non-communicable diseases sub-index, environmental health sub-index and infectious diseases sub-index. Provinces with a similar number of districts and with similar overall scores on the PHDI index showed different levels of relative within-province inequality. Greater within-province relative inequalities were seen in the environmental health and health services provisions sub-indices as compared to other indices.

**Conclusions**: Achieving the goal of ensuring healthy lives and promoting well-being for all at all ages in Indonesia necessitates having a more focused understanding of district-level inequalities across a wide range of public health infrastructure, service, risk factor and health outcomes indicators, which can enable geographical comparison while also revealing areas for intervention to address health inequalities.

## Background

Achieving the Sustainable Development Goal (SDG) of ‘ensuring healthy lives and promoting well-being for all at all ages’, necessitates regular monitoring of inequality in the availability of health-related infrastructure, access to health services, and in health risks and outcomes [1]. Monitoring helps to inform policy, programs, and practices by identifying subpopulations which have inequitable access to services, health risks, and outcomes, and facilitates evaluation of interventions to address these differences [2].

In Indonesia, monitoring subnational regional inequality in key health indicators is particularly relevant because of the structure of the current health system. Following the end of the Suharto era in the late 1990s, Indonesia embarked on political, administrative and fiscal decentralization, with responsibility for major public services like health, education, and infrastructure devolving to local authorities at the district level [3–8]. Despite substantial increases in overall health funding in Indonesia as part of decentralization, evidence indicates variable public expenditure on health, and inequitable service access and quality of health services between districts in Indonesia [4,5,7,9]. Heywood and Choi (2010) for example, reported a reduction in the level of performance of the health system between 2002 and 2007, as measured by changes in the use of maternal and child health services, and substantial variation in these indicators between 10 districts in Central and East Java [4]. Sparrow and colleagues (2017), comparing data from 262 districts across Indonesia between 2004 and 2010 also noted substantial variability between districts in terms of impact of health financing on schemes and health service use [7].

Given these findings, a clearer but also wider sense of the picture in Indonesia was needed. Composite measures combine relevant indicators in a systematic way into an index that summarizes the number of topics of interest as a single numerical value [2,10]. They can be a useful way of facilitating comparisons over time in a given dimension of inequality, like subnational region, to monitor progress toward a health target [2]. The United Nations Development Programme’s Human Development Index (HDI), for example, uses multiple indicators, capturing three dimensions considered relevant to human development: (1) ‘a long and healthy life’; (2) ‘access to knowledge’; and (3) ‘a decent standard of living’, and is used to compare human development between countries and monitor the progress of human development over time, within a country [11]. WHO developed a Universal Health Coverage (UHC) index of essential health services as a means of regularly tracking progress toward UHC–SDG Target 3.8 [10,12]. The UHC index of essential health services combines 16 indicators in a single index which broadly encompass the four domains key to UHC: (1) reproductive, maternal, newborn and child health; (2) infectious diseases; (3) non-communicable diseases; and (4) service capacity and access [12]. As with the HDI, the UHC index of essential services was developed as a means of comparing UHC across countries, but importantly, facilitates the monitoring of progress over time within countries.

In Indonesia, life expectancy was earlier used as a measure of health development in districts. However, this had its limitations in terms of being able to assess interventions in the health sector, and to prioritize across domains of health. Given the particular relevance of monitoring performance of the health sector at subnational level, a composite index was felt to be important to both develop and assess equity-oriented interventions [13]. The Public Health Development Index (PHDI) was developed in 2009, and subsequently mandated in 2010 by national decree to be used to monitor development within and across districts through the analysis and interpretation of simple, easily measurable, credible, and timely indicators across a range of domains: public health infrastructure, services, behavioural risk factors and health outcomes [14]. The aim of this study was to use this composite index to assess various dimensions of public health development across provinces and also to identify within-province inequalities in order to prioritize areas for intervention.

## Methods

We used data from a PHDI report in 2014 which can be freely accessed from National Institute of Health Research and Development, Ministry of Health official website (http://labmandat.litbang.depkes.go.id/images/download/publikasi/IPKM_2013_C3.pdf) [15].

### Development of the PHDI

The PHDI is a composite indicator combining 30 health indicators from across the life course and along the continuum of care. It was adapted from Blum’s work on health and its determinants in a highly consultative process beginning in 2009 [16–18]. Following a legal decree in 2010, the PHDI has been used by the Indonesian Ministry of Health to enable district ranking and intervention development in public health. In 2013, the PHDI was improved with some additions and amendments in indicator selection (see Appendix 1 for details on the development of PHDI in 2013).

### Data source for PHDI indicators

The PHDI and sub-indices in this analysis were derived from the 2013 Basic Health Survey (RISKESDAS) and the 2011 Village Potential Survey (Potensi Desa/PODES). RISKESDAS was the main source for the health indicators; the 2011 yielded indicator related to the health workforce and facilities. RISKESDAS employed a multistaged sampling frame powered to obtain district-level estimates. Over a million respondents were interviewed, with district sample sizes ranging from 656 to 4279. PODES data was a universal sample of all villages in Indonesia. Data for chosen indicators were extracted and Relative Standard Errors (RSEs) calculated for each indicator to assess the stability of the estimates at the district level. Indicators with RSEs higher than 30% were excluded from the analysis. Study data from both sources (RISKESDAS and PODES) were linked using district-level unique identifiers. Indicators were grouped into seven categories: reproductive and maternal health (4 indicators), newborn and child health (6 indicators), infectious diseases (3 indicators), environmental health (2 indicators), non-communicable diseases (6 indicators), health risk behaviors (5 indicators) and health service provision (4 indicators) (see Supplementary Tables 1 and 2 for detailed indicator definitions). Population share was calculated using population census estimates [19].

**Table 1.** Public Health Development Index (overall index and sub-indices): national and province averages, Indonesia, RISKESDAS, 2013.

Province* (by island)	PHDI (overall)	Reproductive and maternal health sub-index	Newborn and child health sub-index	Infectious diseases sub-index	Environmental health sub-index	Non-communicable diseases sub-index	Health risk behavior sub-index	Health services provision sub-index	Number of districts
National average	54.0	47.6	61.1	75.1	54.3	62.7	36.5	38.1	497
**Sumatra**									
Aceh	50.5	43.3	60.4	69.7	42.0	62.6	32.2	43.3	23
North Sumatra	54.2	33.2	60.4	55.0	49.1	38.3	19.2	25.2	33
West Sumatra	54.6	48.6	64.4	77.8	43.6	67.9	30.3	49.9	19
Riau	55.4	47.9	62.4	82.6	50.9	72.8	35.5	35.5	12
Jambi	53.4	46.1	62.4	83.1	43.9	73.1	37.1	28.4	11
South Sumatera	53.0	46.4	62.2	80.7	43.7	70.9	35.4	31.7	15
Bengkulu	53.3	50.6	63.2	79.5	44.4	72.9	33.7	28.6	10
Lampung	54.5	51.4	61.2	83.0	38.9	75.6	35.9	35.3	14
Bangka Belitung	53.6	47.5	66.0	76.0	49.0	57.8	39.6	39.4	7
Riau Islands	60.8	48.8	69.5	80.3	68.5	68.0	38.6	51.9	7
**Java-Bali**									
DKI Jakarta	60.9	51.3	71.7	71.1	83.3	56.2	40.6	51.9	6
West Java	54.6	48.8	67.3	74.4	54.1	60.3	34.6	42.6	26
Central Java	56.3	52.0	65.2	73.8	55.9	63.6	38.3	45.4	35
DI Yogyakarta	57.3	54.1	69.7	76.7	48.0	52.9	40.6	59.3	5
East Java	54.1	49.3	64.7	72.9	54.3	58.6	36.4	42.7	38
Banten	56.8	45.9	66.7	73.7	69.7	65.5	34.2	42.0	8
Bali	65.0	59.0	70.6	78.6	72.7	66.6	45.6	62.2	9
**Nusa Tenggara Islands**									
West Nusa Tenggara	52.4	47.3	62.6	72.6	41.6	66.0	32.2	44.3	10
East Nusa Tenggara	46.2	33.1	56.5	61.8	30.8	64.1	37.3	40.0	21
**Kalimantan**									
West Kalimantan	51.4	43.4	57.2	82.3	41.3	71.0	39.6	25.2	14
Central Kalimantan	50.5	24.1	58.6	47.4	32.6	42.4	23.2	16.6	14
South Kalimantan	48.6	27.1	59.0	41.0	34.7	27.3	16.9	14.1	13
East Kalimantan	57.6	32.7	66.8	61.2	59.8	28.1	27.8	36.8	14
**Sulawesi**									
North Sulawesi	54.3	34.3	66.1	46.1	46.4	20.5	27.7	29.3	15
Central Sulawesi	48.9	20.1	60.2	32.3	42.3	19.8	15.9	16.6	11
South Sulawesi	52.4	22.2	62.1	34.6	42.0	15.6	25.9	40.0	24
Southeast Sulawesi	51.6	21.4	59.8	47.5	42.6	38.5	24.6	16.9	12
Gorontalo	51.1	31.9	61.2	44.5	30.9	28.1	21.8	30.5	6
West Sulawesi	49.8	37.6	57.8	73.9	44.9	67.0	41.1	31.6	5
**Maluku Islands**									
Maluku	49.4	32.7	57.7	75.8	53.3	66.2	36.9	25.3	11
North Maluku	49.6	37.6	58.9	80.0	46.3	66.8	37.8	24.2	9
**Papua**									
West Papua	49.7	34.1	58.1	77.9	44.7	69.1	38.5	31.9	11
Papua	43.9	32.1	56.7	66.0	25.0	70.9	33.5	27.8	29

*Ordered according to Indonesian government codes of geographical proximity.

**Table 2.** Relative within-province inequality (measured by Weighted Index of Disparity) in Public Health Development Index (overall index and sub-indices): Indonesia, RISKESDAS, 2013.

Province (by island)	PHDI (overall)	Reproductive and maternal health sub-index	Newborn and child health sub-index	Infectious diseases sub-index	Environmental health sub-index	Non-communicable diseases sub-index	Health risk behavior sub-index	Health services provision sub-index	Number of districts
**Sumatra**									
Aceh	9.8	11.8	9.5	10.4	32.8	9.2	12.0	16.9	23
North Sumatra	9.0	20.2	8.3	10.9	33.0	21.5	14.0	39.8	33
West Sumatra	8.2	7.2	9.1	6.5	27.3	5.6	14.8	11.6	19
Riau	10.3	11.3	6.5	4.3	32.9	6.4	9.9	33.0	12
Jambi	6.5	9.4	7.9	4.9	27.2	8.6	12.7	26.4	11
South Sumatra	7.0	9.3	6.9	6.1	33.5	9.9	12.0	36.4	15
Bengkulu	5.5	7.1	6.4	5.4	21.9	7.0	13.3	20.1	10
Lampung	5.2	5.9	5.7	4.7	18.3	3.9	9.7	25.4	14
Bangka Belitung	4.3	5.2	7.9	5.5	24.0	9.8	12.8	14.3	7
Riau Islands	5.2	1.1	2.6	2.3	20.9	6.8	4.8	2.9	7
**Java-Bali**									
DKI Jakarta	2.6	4.1	4.7	6.3	5.9	13.7	4.7	6.6	6
West Java	7.7	8.0	5.9	6.5	29.0	11.5	8.8	14.1	26
Central Java	5.7	6.4	8.1	6.2	15.8	8.4	8.4	13.8	35
DI Yogyakarta	1.4	5.0	3.9	1.5	2.7	6.9	4.8	6.6	5
East Java	6.7	6.1	7.8	6.2	23.6	12.1	9.0	11.1	38
Banten	6.8	14.0	7.5	4.7	19.8	9.0	12.0	11.4	8
Bali	6.7	8.2	6.8	6.8	11.3	9.0	6.5	12.4	9
**Nusa Tenggara Islands**									
West Nusa Tenggara	5.1	8.8	6.7	6.2	25.5	4.4	9.3	10.6	10
East Nusa Tenggara	12.2	20.5	14.3	13.7	44.0	10.9	14.6	18.9	21
**Kalimantan**									
West Kalimantan	8.6	13.3	12.4	4.0	25.8	7.3	19.3	34.7	14
Central Kalimantan	9.4	14.0	10.4	9.3	45.9	10.2	17.9	40.0	14
South Kalimantan	11.1	18.9	9.4	16.1	40.6	13.2	25.1	67.9	13
East Kalimantan	6.9	7.8	5.5	5.7	18.5	21.8	6.1	31.1	14
**Sulawesi**									
North Sulawesi	7.7	9.1	7.8	14.4	23.7	23.8	13.3	38.6	15
Central Sulawesi	10.3	21.8	8.5	24.1	29.0	24.6	23.7	43.0	11
South Sulawesi	8.0	15.8	8.1	16.7	33.4	42.1	9.4	20.6	24
Southeast Sulawesi	7.8	29.6	8.7	13.5	27.0	19.6	9.6	56.7	12
Gorontalo	4.6	15.7	4.4	11.0	29.3	19.7	20.5	16.9	6
West Sulawesi	4.5	6.8	3.2	16.5	22.4	11.6	6.1	22.7	5
**Maluku Islands**									
Maluku	9.2	13.7	12.3	8.6	25.4	6.3	10.8	32.1	11
North Maluku	10.6	16.8	14.8	6.8	29.6	11.6	11.4	29.1	9
**Papua**									
West Papua	4.7	14.0	0.0	6.8	30.5	14.3	7.9	11.8	11
Papua	18.8	35.5	24.8	19.8	84.9	8.8	16.2	36.5	29

### Calculation of the PHDI

The PHDI was calculated following nine steps which are described at length in Appendix 1 and detailed elsewhere [15,17]. First, values for 30 health indicators comprising the PHDI were calculated. In order to ensure comparability of different indicator types and indicator scales, adverse indicators (where a lower value is desirable) were rescaled to ensure they have the same direction as favorable indicators (where a higher value is desirable). Theoretical values for each indicator were obtained based on expert consensus, especially for disease prevalence (where the lowest actual values was determined to be the worst theoretical value of the indicator). Then, indicators were normalized to range from 0 (worst) to 1 (best). Individual indicators were assigned weights based on expert consensus regarding the size of population affected, the degree of impact it had on health, the felt urgency in addressing this indicator, and the degree of complexity anticipated in addressing the underlying health issue. Weights were then assigned proportionately for each indicator, for sub-indices and then the overall PHDI score at various levels (districts, province, national). Finally, index values were scaled to be expressed as percentages (i.e. out of 100).

### Data analysis

Estimates for the overall index and sub-indices were calculated at the national, provincial and district level. Figures were calculated for 33 provinces and 497 districts in 2013 (the year of data collection), based on district and category definitions provided by the Ministry of Home Affairs using SPSS version 17 [20]. Given that the mandate of the PHDI was to assess district-level differences in public health development within Indonesia, we additionally used a summary measure of inequality to look at inequalities across districts within provinces.

Thus, a relative measure of inequality, the Weighted Index of Disparity (IDISW), was used to assess the level of inequality for the indices within provinces, i.e across districts. The IDISW shows the average proportional difference between each district and the province average. It is calculated as the weighted sum of absolute differences between the district estimates $y_{i}$ and the province average μ, divided by the province average μ and multiplied by 100. Absolute differences are weighted by each district’s population shared $p_{i}$.

$$Weighted\;Index\;of\;Disparity=\frac{\sum p_{i}*\left|y_{i}-\mu \right|}{\mu }*100$$

The IDISW is a relative measure of inequality that takes into account the PHDI level and the population share of each of the districts within a province as well as the PHDI level of that province [13]. It is therefore well suited to our purpose of examining inequalities across non-ordered population subgroups (i.e. districts) that may have varying population sizes – allowing us to examine the magnitude of inequality in comparable units across provinces. Values of the IDISW range from 0 upwards; the closer the value is to 0, the lower the level of inequality (which is desirable). Disaggregated estimates for districts were prepared using SPSS version 17 and entered into the Health Equity Assessment Toolkit Plus (HEAT Plus), which generated IDISW estimates for each province [21].

## Results

### National and province-level PHDI scores

**Figure 1.**
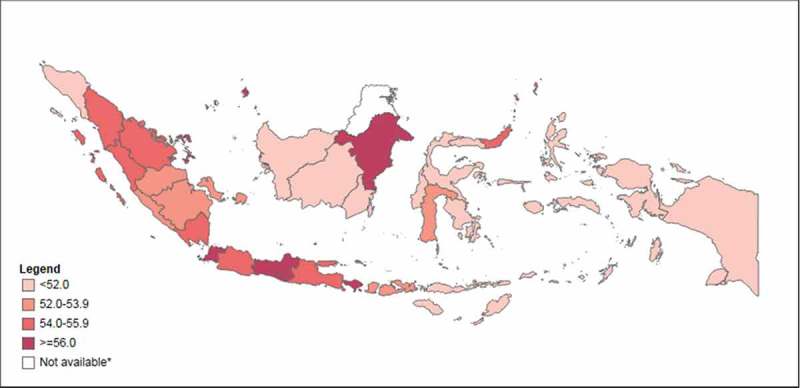
Public Health Development Index (overall), in 33 provinces, Indonesia, 2013. Note: Data are not available for North Kalimantan province, which was created in 2012.

Table 1 shows the national and province averages of the overall index and sub-indices; Figure 1 shows provincial PHDI scores on a map. The national PHDI score was 54.0 (out of 100). Among the sub-indices, the highest national average was reported for the infectious diseases sub-index (75.1), followed by the non-communicable diseases sub-index (62.7) and the newborn and child health sub-index (61.1). The environmental health sub-index had a national average of 54.0, and the reproductive and maternal health sub-index was 47.6. The lowest national averages were observed for the health services provision sub-index (38.1) and the health risk behavior sub-index (36.5).

Inequalities between provinces were observed for all indices. Bali had the best province PHDI score of 65.0 while Papua performed the worst of all provinces with a PHDI score of 43.9. Large variations were observed for the non-communicable diseases sub-index (ranging from 15.6 for South Sulawesi to 75.6 for Lampung), environmental health sub-index (ranging from 25.0 for Papua to 83.3 for DKI Jakarta) and infectious diseases sub-index (ranging from 32.3 for Central Sulawesi to 83.1 for Jambi). Central Sulawesi had the poorest reproductive and maternal health and health risk behavior sub-index scores (province averages of 20.1 and 15.9, respectively), while Bali performed best (province averages of 59.0 and 45.6, respectively). Bali also had the highest score for the health services provision sub-index (62.2), while South Kalimantan had the lowest score on this index (14.1). The smallest variations were observed for the newborn and child health sub-index, with scores ranging from 65.5 in East Nusa Tenggara to 71.7 in DKI Jakarta.

Provinces in the western part of the country tended to report higher overall PHDI scores than provinces in the east. Looking at the sub-indices, Bali, DI Yogyakarta and DKI Jakarta tended to perform well (among the top five provinces for at least four out of the seven sub-indices), while Central Kalimantan, Central Sulawesi, Gorontalo and South Kalimantan tended to perform poorly (among the bottom five provinces for at least four out of the seven sub-indices).

### Within-province inequalities in PHDI scores

**Figure 2.**
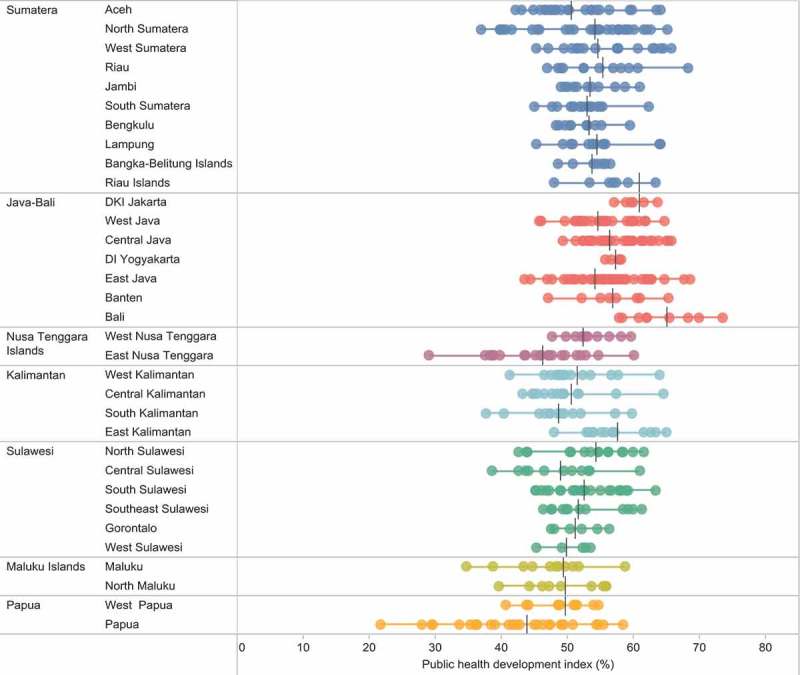
Public Health Development Index (overall), by district, in 33 provinces, Indonesia, 2013.

Inequalities were apparent between districts within each province. Looking at PHDI values, a few provinces reported especially large variations between districts (see Figure 2). For example, in Papua, the province average was 43.9, yet four districts in this province reported an index below 30 (Intan Jaya, Lanny Jaya, Mamberamo Raya, and Tolikara districts), while Jayapura city reported an index of 58.4. In East Nusa Tenggara the overall index ranged from 29.0 in Manggarai Timur district to 60.1 in Kupang city.

**Figure 3.**
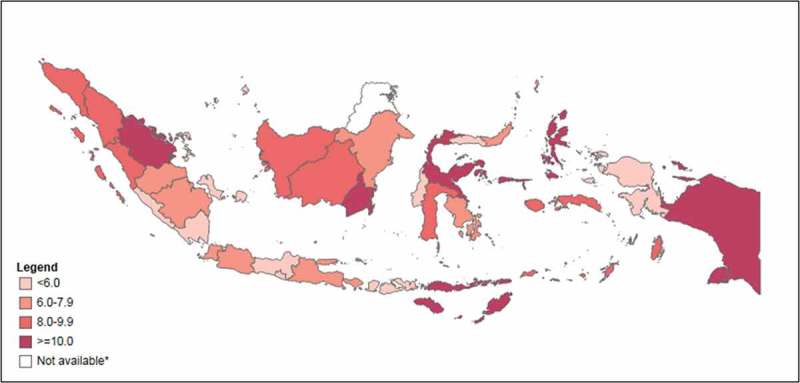
Relative within-province inequality in Public Health Development Index measured as Weighted Index of disparity, in 33 provinces, Indonesia, 2013. Note: Data are not available for North Kalimantan province, which was created in 2012.

**Figure 4.**
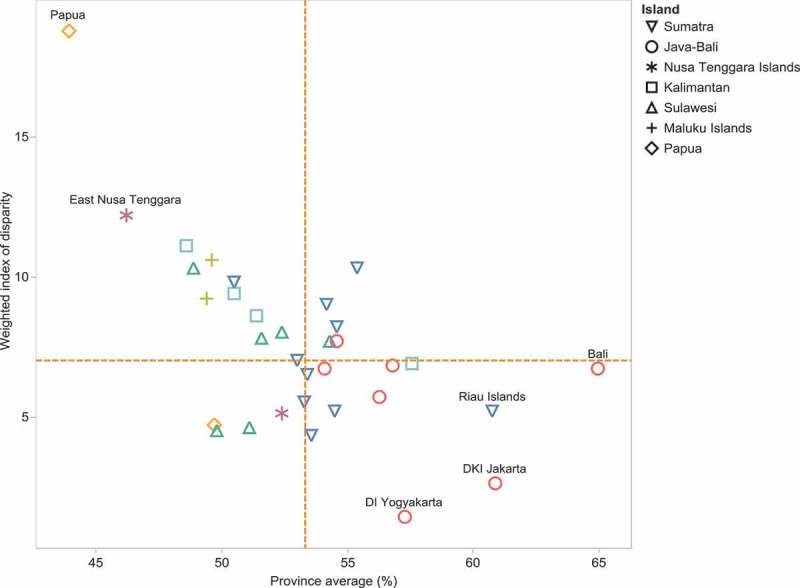
Public Health Development Index (overall index): province average and relative within-province inequality, Indonesia, RISKESDAS, 2013. Note: Dashed orange lines indicate the median values.

Table 2 presents the level of relative inequality by districts, measured as IDISW, within each province for the overall index and the seven sub-indices; Figure 3 shows IDISW values across Indonesia on a map. Provinces with a similar number of districts showed different levels of relative within-province inequality. For example, neighboring provinces West Papua and North Maluku both reported an overall PHDI of 50, yet relative within-province inequality was more than twice as great in North Maluku compared with West Papua (IDISW = 10.6 compared with IDISW = 4.7) (see Figure 4). Similarly, Lampung and Riau both had an overall PHDI of 55, yet relative within-province inequality was almost twice as high in Riau compared with Lampung (IDISW = 10.3 and IDISW = 5.2, respectively). DKI Jakarta and Riau Islands were both among the provinces with the highest overall PHDI (61), yet relative within-province inequality was twice as high in the Riau Islands compared with DKI Jakarta (IDISW = 5.2 compared with IDISW = 2.6).

**Figure 5.**
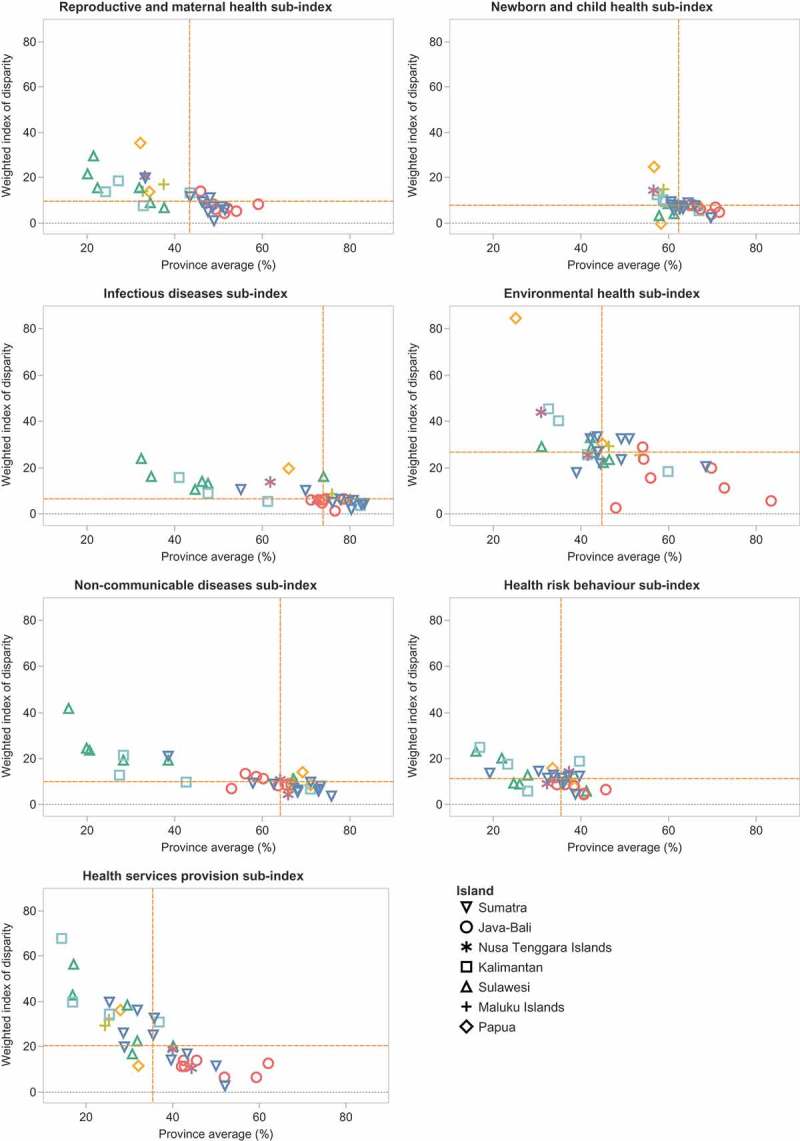
Sub-indices of Public Health Development Index: province average and relative within-province inequality, Indonesia, RISKESDAS, 2013. Note: Dashed orange lines indicate the median values.

Considering the level of relative within-province inequality alongside the province average, different patterns were evident for different sub-indices (see Figure 5). The highest levels of relative within-province inequality could be observed for the environmental health and health services provision sub-index (median IDISW values of 27 and 21, respectively), alongside lower province averages (median province average below 50). The infectious diseases, non-communicable diseases and newborn and child health indices tended to have lower relative within-province inequalities (median IDISW below 10) and higher province averages (median province average above 60). Relative within-province inequalities were also low for the reproductive and maternal health sub-index and province averages were moderate. The health risk behavior reported moderate levels of relative within-province inequality, but the lowest province averages.

## Discussion

Patterns of inequality in the PHDI were observed across and within provinces of Indonesia: echoing findings from other studies which have found geographic inequalities in health related indicators in the country [4,9,22–24]. According to the 2015 Indonesia Health Profile, the proportion of the population living in poverty in the province with the lowest PHDI score, Papua (28.4%), was over five times that of the province with the highest PHDI score: Bali (5.25%), suggesting that the PHDI score shows basic concurrent validity with the broader patterns of economic inequality in the country [25]. This would have to be directly tested in a further study with more indices and measures.

Moreover, we found that the eastern provinces of the country had low PHDI index levels and high within province inequalities. More specifically, the provinces of South Kalimantan, East Nusa Tengara and Papua had the lowest overall PHDI values and highest levels of IDISW. These provinces are constrained by an intersection of poverty and geographical barriers (due to hilly and island terrain) [22,26,27]. This is the least desirable result from the perspective of public health and development and of inequality and suggests that these are geographies for priority action to improve health services while also addressing inequalities across districts.

Previous studies have found that decentralization has yet to yield substantial gains in health system performance, specifically in relation to reproductive, maternal and child health [4,24]. In this study, the provinces in the eastern regions of Sulawesi and Kalimantan reported the lowest PHDI sub-scores for reproductive and maternal health, as well as newborn and child health. Yet in other regions, like many provinces in the Java-Bali and Sumatra regions, PHDI sub-scores were above the national average and inequalities among the lowest in the country, suggesting path dependency on local factors that may predate decentralisation. More research is required to answer this question.

In addition, the challenges of non-communicable diseases loom large in the country [28]. From an equity perspective, substantial within-province relative inequality in the NCD sub-index was seen in Sulawesi, which happens to have the lowest sub-index scores for NCDs as well. Moreover, it is in Sulawesi and Kalimantan that the greatest relative inequalities are seen in the health services sub-indices, an observation which is borne out by other research as well [23,29]. In contrast, Java-Bali, with the highest sub-index scores for health services (as well as relatively lower levels of within-province inequality) has attracted a great deal of health human resources (including specialists), in part due to laxity in regulation and the draw of the private sector [30].

As these examples demonstrate, the use of the PHDI’s seven sub-indices can help indicate where different regions of the country may place emphasis to address inequality. The highest values for relative inequality were seen in the environmental health (maximum value: 84.9) and health services (maximum value: 67.9) sub-indices. These are clearly areas where greater policy emphasis must be placed. In addressing the environmental health sub-index, interventions to increase access to safe water and sanitation are desirable. Indonesia’s Ministry of Health has a mid-term development plan which has placed a target of universal access to improved water and sanitation, safe drinking water as well as progressive elimination of slum habitation and open defecation [31]. These are ambitious targets with strong equity implications given that, for instance, in 2015, less than half of all households (47%) had access to improved sanitation [32]. Action in this domain is being pursued through a number of approaches, including community based total sanitation (Sanitasi Total Berbasis Masyarakat – STBM), which is already underway in the country [33]. Future programming may emphasize action in provinces where inequalities are greater (Papua, North Kalimantan, South Kalimantan, and East Nusa Tenggara) to ensure universalization, particularly since neighboring provinces in the same islands have higher scores and may offer lessons for implementation.

Health service delivery has been a challenge for a long time in Indonesia, as in many other countries [23,24]; shortages in these building block-type indicators really lie at the heart of other inequalities. For example, the number of general practitioners per 100,000 population ranged from 8.8 to 155.5 in 2013 [34]. Further, Indonesia’s 2011 health facility survey showed that only 18.6% of health centers were able to provide basic emergency care, known by the acronym PONED. Of PONED-certified health centers, only 78% were able to provide 24-hour service, largely due to the inability of facilities in eastern regions such as Papua, Maluku and East Nusa Tenggara to do so [35]. Indonesia has introduced a program for accreditation of hospitals and health centers to remedy this [36]. Furthermore, the Nusantara Sehat program has been launched to improve a quality health service for people in remote and border areas [37]. Carrying out a similar analysis longitudinally may help assess whether Nusantara Sehat has been able to reduce inequalities in these regions.

The PHDI is among many other indices used globally for sorting and priority-setting. While other indices (e.g. Human Development Index [11], Universal Health Coverage index [12], Healthcare Access and Quality Index [38], Urban Health Index [39], Gender Development Index [40]), provide useful, globally comparable information, the PHDI specifically allows for subnational analysis to facilitate district-level comparisons and intervention planning. It was developed in-country with the political buy-in of district level authorities with an aim to create a culture of ‘competition’ to improve health development across districts. The PHDI has enabled focused programmatic and fiscal attention on aspects of health service, such as increasing access to health workers and health facilities in villages, partnership between village midwife and traditional birth attendances, direct budget allocation for each village and improving district infrastructure for transportation and communication. It has been used to monitor health progress at district level and select priority districts for integrated interventions. A mentoring program has been implemented since 2012 for low performing districts, involving in-kind assistance to local governments and coordination between them and central government actors [41].

These strengths notwithstanding, the PHDI is not without limitations. The selection of indicators by experts has been an admitted challenge, limited by the availability and quality of data sources. Slightly different indicators could change – at least somewhat – the properties of the index. As with all such exercises, moreover, aspects of health and development that are not amenable to being measured as indicators cannot figure in the index. Given the process followed, however, every effort was made to ensure that the validity of the results was high [14,42]. Similar exercises – based on expert selection and ranking of indicators – has been carried out in other countries in the region, although district representativeness is a unique feature of the Indonesian PHDI.

To conclude, achieving the goal of ensuring healthy lives and promoting well-being for all at all ages in Indonesia necessitates having a more focused understanding of district-level inequalities across a wide range of public health infrastructure, service, risk factor and health outcomes indicators, which can enable comparison while also revealing areas for intervention to address subnational regional inequality in health. The PHDI has facilitated this and offers promise in helping the country advance on its path towards achieving the SDGs.

## Paper context

Achieving the SDG of ‘ensuring healthy lives and promoting well-being for all at all ages’ necessitates regular monitoring of inequality of health-related infrastructure, access to health services, health risks and outcomes. This paper presents the Indonesian Public Health and Development Index and its sub-indices at the national, provincial and district levels, reports within province inequalities in the index and its sub-indices, and reveals topical and geographic areas for intervention to address health inequalities.

## Appendix 1. Formulation of the public health development index (PHDI) 2013

The formulation of PHDI explained below was extracted and from the following citation: The 2013 PHDI report in the National Institute of Health Research and Development, Ministry of Health official website (http://labmandat.litbang.depkes.go.id/images/download/publikasi/IPKM_2013_C3.pdf).

The PHDI composite index was formulated as follows:

1. *Indicator selection*: The PHDI and sub-indices were derived from the 2013 Basic Health Survey (RISKESDAS) and the 2011 Village Potential Survey (Potensi Desa/PODES). RISKESDAS was the main source for the health indicators. The 2011 PODES provided data for part of the PHDI (overall) indicator related to the health workforce and facilities. RISKESDAS employed a multistaged sampling frame powered to obtain district-level estimates. Over a million respondents were interviewed, with district sample sizes ranging from 656 to 4279. PODES data was a universal sample of all villages in Indonesia. Data for chosen indicators were extracted and Relative Standard Errors (RSE) calculated for each indicator to assess the stability of the estimates at the district level. Indicators with RSE higher than 30% were excluded from the analysis. Study data from both sources (RISKESDAS and PODES) were linked using district-level unique identifiers. The 30 selected indicators were grouped into seven categories: reproductive and maternal health (4 indicators), newborn and child health (6 indicators), infectious diseases (3 indicators), environmental health (2 indicators), non-communicable diseases (6 indicators), health risk behaviors (5 indicators) and health service provision (4 indicators)
2. *Empirical value assignment for comparability*: In order to ensure comparability of different indicator types and indicator scales, adverse indicators (where a lower value is desirable) were rescaled to ensure they have the same direction as favorable indicators (where a higher value is desirable).
  On the coverage variables, the empirical values were the exact value of the proportion or percentages. In variables of the disease prevalence, the empirical values were calculated as in equation 1.
  Equation 1. Calculation empirical values
  (1) Prevalenceempiricalvalues=100−theprevalencevalue

1. *Theoretical value assignment*: we define maximum and minimum theoretical values for the standard of each indicator. Lower and upper bounds for these theoretical values were based on current Indonesian health status. Theoretical values for coverage ranged from 0 (worst) and 100 (best). For disease prevalence, our experts agreed on the use of the lowest actual value as the worst theoretical value possible whereas the best value is considered as equal to 100.
2. *Indicator normalization*: all indicators were normalized to range from 0 (worst) to 1 (best) using equation 2.
  Equation 2. Normalizing the indicators

(2) $$Normalization\;=\;\frac{Empirical\;values-minimum\;theoretical\;value}{maximum\;theoretical\;value-minimum\;theoretical\;value}$$

1. *Weighting factor assignment*: we define the weighting factor for each indicator. The weighting factors were based on expert consensus, each indicator was weighted by 3, 4 or 5 based on population affected, impact on health status, urgency and complexity of response. An example of the weighting process is given below in Table A2; overall weights applied are indicated in Table A1.
  **Table A1.** Defining weighting factor for each indicator.
  		Component				
  Indicators	Score base	Size of population affected	Impact on health status	Urgency	Complexity of response	Weight (2 + 3 + 4 + 5 + 6)
  (1)	(2)	(3)	(4)	(5)	(6)	(7)
  Prevalence of underweight and severe underweight children under five years of age	1	1	1	1	1	5
  Prevalence of mental health disorder	1	0	1	1	1	4
  Proportion exhibiting safe hand washing behavior	1	0	1	1	0	3
2. *Sub-index weight adjustment*: We grouped indicators into sub-indices and calculated sub-index weights using an arithmetic approach. For example, the reproductive and maternal health sub-index was derived from 3 indicators, all of which had a 5 point weight. The proportion of weight was calculated by dividing the weight by the total weight (equation 3). The proportion of weight for the reproductive and maternal health sub-index was 5/15.
  Equation 3. Calculation of indicator weight under each sub-index

(3) $$\begin{array}{c} Proportion\;of\;weight=\frac{weight}{Total\;weight} \end{array}$$

1. *Indicator weight adjustment*: We calculated an index score for each indicator, which was the normalization score multiplied by that indicator’s weight proportion.
2. *Sub-indices calculated with weights applied*: We calculated sub-indices by the sum of the weighted individual indicators for a sub index.
3. *Overall PHDI index calculated with weights applied*: we calculated the overall index by combining all sub-indices with equal weights applied (see Table A2).

## Appendix

**Table A2.** Example of PHDI calculation for district ‘X’.

				Theoretical values						
No.	Indicators	Values	Empirical values	Min	Max	Normalization	Weight	Proportion of weight	Index of indicators	Overall index and sub-indices
(a)	(b)	(c)	(d)	(e)	(f)	(i)	(j)	(k)	(l)	(m)
	Public Health Development Index (PHDI)									0.61
	Reproductive and maternal health						15			0.56
1	Proportion of long-term method contraceptive use	19.5	19.5	0.0	100.0	0.2	5	0.33	0.07	
2	Proportion of antenatal care	63.8	63.8	0.0	100.0	0.6	5	0.33	0.21	
3	Prevalence of chronic malnutrition among women	11.5	88.5	25.3	100.0	0.8	5	0.33	0.28	
	Newborn and child health						26			0.67
4	Prevalence of underweight and severe underweight in children under five years of age	14.9	85.1	52.4	100.0	0.7	5	0.19	0.13	
5	Prevalence of stunting and severe stunting in children under five years of age	34.0	66.0	29.6	100.0	0.5	5	0.19	0.10	
6	The prevalence of obesity in children under five years of age	17.0	83.1	19.6	100.0	0.8	4	0.15	0.12	
7	Proportion of children under 5 years of age attending monthly growth monitoring in the last 6 months	66.0	66.0	0.0	100.0	0.7	4	0.15	0.10	
8	Proportion of complete basic immunization	48.1	48.1	0.0	100.0	0.5	4	0.15	0.07	
9	Proportion of first neonatal visits	95.1	95.1	0.0	100.0	1.0	4	0.15	0.15	
	Infectious diseases						13			0.78
10	Prevalence of pneumonia	1.9	98.1	80.4	100.0	0.9	5	0.38	0.35	
11	Prevalence of diarrhea among children under five years of age	12.4	87.7	35.4	100.0	0.8	4	0.31	0.25	
12	Prevalence of ARI among children under five years of age	33.9	66.1	16.2	100.0	0.6	4	0.31	0.18	
	Environmental health						6			0.57
14	Proportion of households with access to safe sanitation facilities	69.3	69.3	0.0	100.0	0.7	3	0.50	0.35	
13	Proportion of households with access to clean water	45.3	45.3	2.0	100.0	0.4	3	0.50	0.22	
	Non-communicable diseases						27			0.63
15	Prevalence of hypertension	15.7	84.3	58.4	100.0	0.6	5	0.19	0.12	
16	Prevalence of injury	6.4	93.6	74.8	100.0	0.7	5	0.19	0.14	
17	Prevalence of diabetes mellitus	1.9	98.1	95.2	100.0	0.6	5	0.19	0.11	
18	Prevalence of mental health disorder	6.5	93.5	51.6	100.0	0.9	4	0.15	0.13	
19	Prevalence of central obesity	32.2	67.8	39.4	100.0	0.5	4	0.15	0.07	
20	Prevalence of dental and mouth problems	28.8	71.2	48.5	100.0	0.4	4	0.15	0.07	
	Health risk behaviors						16			0.42
21	Proportion of daily smoking behavior	27.2	72.8	55.9	100.0	0.4	4	0.25	0.10	
22	Proportion of hand washing behavior	70.2	70.2	1.3	100.0	0.7	3	0.19	0.13	
23	Proportion of open defecation	88.1	88.1	6.7	100.0	0.9	3	0.19	0.16	
24	Proportion of inactive physical activity	20.8	20.8	5.5	100.0	0.2	3	0.19	0.03	
25	Proportion of proper teeth brushing	1.4	1.4	0.0	100.0	0.0	3	0.19	0.00	
	Health services provisions						20			0.63
26	Proportion of institutional delivery	97.6	97.6	0.0	100.0	1.0	4	0.20	0.20	
27	Proportion of sub-districts with sufficient number of GPs	20.0	20.0	0.0	100.0	0.2	5	0.25	0.05	
28	Proportion of village with sufficient number of health posts	82.4	82.4	0.0	100.0	0.8	4	0.20	0.16	
29	Proportion of midwife sufficiency	13.7	13.7	0.0	100.0	0.1	3	0.15	0.02	
30	Proportion of health insurance ownerships	97.7	97.7	0.2	100.0	1.0	4	0.20	0.20	
